# Yin Huo Tang, a traditional Chinese herbal formula, relives ovariectomy and empty bottle stimulation-induced menopause-like symptoms in mice

**DOI:** 10.3389/fendo.2022.994642

**Published:** 2022-10-19

**Authors:** Yang Ye, Bo Zhang, Yan Li, Hong-Dan Xu, Xiu-Min Liu, Shu-Ming Huang, Rui Wang, Dong Li

**Affiliations:** ^1^ Department of Traditional Chinese Medicine, Peking University Third Hospital, Beijing, China; ^2^ Department of Neuroscience, Institute for Chinese Medicine, Heilongjiang University of Chinese Medicine, Harbin, China; ^3^ Department of Integrated Traditional Chinese and Western Medicine, College of Medicine, Yangzhou University, Yangzhou, China; ^4^ Department of Pharmacy, Wuxi Higher Health Vocational Technology School, Wuxi, China; ^5^ National Institute on Drug Dependence and Beijing Key Laboratory of Drug Dependence, Peking University, Beijing, China; ^6^ Research Institute of Medicine and Pharmacy, Qiqihar Medical University, Qiqihar, China

**Keywords:** menopause, Yin Huo Tang, ovariectomy, empty bottle stimulation, estradiol

## Abstract

**Background:**

Yin Huo Tang (YHT), a traditional Chinese herbal formula, is effectively used for the clinical treatment of menopause-like symptoms in China. This study aimed to investigate its efficacy on menopause-like symptoms in mice using behavioral tests and histopathological assessment, and to determine its possible mechanism of action based on network pharmacology.

**Methods:**

Liquid chromatography-mass spectrometry (LC-MS) technology was used to identify the potential active ingredients of YHT. In mice, menopause-like symptoms were induced by combination of bilateral ovariectomy and empty bottle stimulation. The mice were then treated with the YHT aqueous extract for three weeks. Behavior, sleep state, body weight, organ index, and histomorphology were analyzed separately. Additionally, network pharmacology and molecular docking were used to predict the mechanisms underlying the action of YHT. Finally, serum estradiol was quantified to preliminarily verify the results of network pharmacology.

**Results:**

YHT not only improved the behavior of mice (attack and explore behavior reduced; modify behavior increased) but also ameliorated the sleep state (sleep time increased and incubation time reduced). YHT reduced body weight, increased uterine weight, and improved the histomorphology of some organs. Network pharmacology and molecular docking analyses revealed that the estrogen signaling pathway might play a key role in attenuating menopause-like symptoms. Furthermore, YHT treatment reversed the reduction in serum estradiol levels.

**Conclusions:**

YHT alleviates menopause-like symptoms in a mouse model, providing a rationale for using it as a potential therapeutic strategy.

## Introduction

Menopause is the moment women stop menstruating, defined as 12 consecutive months following the last menstrual period. The average age at which women experience menopause is 45–55 years (1). Generally, menopause occurs with physical symptoms (hot flashes, sweating, and poor circulation), emotional symptoms (depressive mood, lack of energy, concentration difficulties, sleep disorders, and emotional instability), and organic disorders (local urogenital atrophy, skin atrophy, and development of osteoporosis) with ample variations (2). The pathogenesis of the menopausal syndrome is mainly associated with a decrease in estrogen levels (3). Hormone replacement therapy (HRT) is an effective treatment method for the menopausal syndrome, which relieves menopausal discomfort and improves the quality of life; however, the use of HRT is restricted by its potential adverse effects (4, 5). Thus, finding an effective treatment method with fewer side effects is important for women with menopausal syndrome.

In China, traditional Chinese medicine (TCM) has become an important therapy for women with menopausal syndrome. Yin Huo Tang (YHT), a classic Chinese medicine formula, was originally recorded in Bian Zheng Lu, which was an ancient Chinese medical book written by Chen Shiduo during the Qing Dynasty. This formula consists of five commonly used herbs: 1) Rehmannia glutinosa (Gaetn.) Libosch. ex Fisch. et Mey. (Shu Dihuang); 2) Morinda officinalis How. (Ba Jitian); 3) Ophiopogon japonicus (L.f.) Ker-Gawl. (Mai Dong); 4) Poria cocos(Schw.)Wolf (Fu Ling); 5) Schisandra chinensis (Wu Weizi). YHT has been clinically used to treat patients with Yin deficiency syndrome such as female menopause syndrome (6–8). In an animal study, YHT demonstrated protective effects against postmenopausal depression and the SIRT1/PGC-1α pathway may be involved in this action (9). The therapeutic potential of YHT requires further confirmation, whereas its possible mechanism of action requires further exploration in animal models.

In this study, we investigated the pharmacological effects of YHT in a mouse model of menopausal syndrome. We assessed the behavior, sleep state, body weight, and organ index, and analyzed the histomorphology and serum estradiol levels in mice. Moreover, we employed network pharmacology and molecular docking to reveal the possible mechanisms underlying the protective effects of YHT. The results of this study provide new insights into this field and provide a scientific basis for the application of YHT in menopausal syndrome.

## Methods

### Animals

In total, 155 female ICR mice were employed at the age of five weeks and the study was performed in a randomized and blinded manner. Animals were housed at an ambient temperature of 22°C with a relative humidity of 60% and a 12 h light-dark cycle. They were provided water ad libitum, except during the testing period. Food was provided ad libitum throughout the experiments. This study was conducted in strict accordance with the local institutional guidelines for animal experimentation. The experimental protocol was approved by the Animal Care and Ethics Committee of the Heilongjiang University of Chinese Medicine. During experiments, all efforts were made to humanize the experiments for animals.

### YHT herbal materials

Dried YHT, consisting of five herbal components (**Table 1**) was purchased from Beijing Tongrentang Pharmaceutical (Harbin, China). We slightly adjusted the doses of the herbs used in this study, as needed. The crude individual herbal components of YHT were cut into small pieces and then mixed at a ratio of 3:2:2:1:1. They were then suspended in distilled water for two hours to soften the raw materials and facilitate the extraction of water-soluble ingredients in subsequent steps. The herbs were boiled for one hour and passed through filter paper. The process lasted three times. The recommended daily intake of YHT is 45 g/day. Converting the clinically equivalent dose for a 70 kg adult to that for a 20 g mouse by body surface area ratio, the reference dose for mice was 5.85 g/kg/day (low dose in the study) (10). The filtrate was condensed to the required concentrations and stored at 4°C for later use.

**Table 1:** Contents of Yin Huo Tang.

Chinese name	Botanical name	Common name	Genus	Family	Weight (g)	Part used
Shu Dihuang	Rehmannia glutinosa (Gaert.) Libosch. ex Fisch. et Mey.	dried rehmannia root	Rehmannia	Scrophulariaceae	15	Root
Ba Jitian	Morinda officinalis How.	morinda officinalis	Morinda	Rubiaceae	10	Root
Mai Dong	Ophiopogon japonicus (L.f.) Ker-Gawl.	radix ophiopogonis	Ophiopogon	Liliaceae	10	Root
Fu Ling	Poria cocos(Schw.) Wolf	tuckahoe	Poria	Polyporaceae	5	Sclerotia
Wu Weizi	Schisandra chinensis	fruit of Chinese magnoliavine	Schisandra	Magnoliaceae	5	Fruit

### LC-MS analysis of YHT

Liquid chromatography-mass spectrometry (LC-MS) analysis was performed using an ACQUITY UHPLC system (Waters Corporation, Milford, USA) coupled with an AB SCIEX Triple TOF 5600 System (AB SCIEX, Framingham, USA). The concentration of YHT analyzed was 0.15 g/mL (standard concentration: 45 g YHT/300 mL water). An ACQUITY UPLC HSS T3 column (2.1×100 mm, 1.8 μm) was employed in both the positive and negative modes. The binary gradient elution system consisted of (A) water (containing 0.1% formic acid, v/v) and (B) acetonitrile (containing 0.1% formic acid, v/v), and separation was achieved using the following gradient: 0 min, 5% B; 2 min, 5% B; 4 min, 25% B; 8 min, 50% B; 10 min, 80% B; 14 min, 100% B; 15 min, 100% B; 15.1 min, 5%; 16 min, 5%B. The flow rate and the injection volume were 0.35 mL/min and 2 μL, respectively. The column temperature was maintained at 45°C, and the samples were kept at 4°C. Data acquisition was performed in the full scan mode (m/z range from 100 to 1000) combined with the IDA mode. The MS parameters were as follows: ESI ion source temperature: 550°C (+) and 550°C (−); ion spray voltage: 5500 V (+) and 4500 V (−); curtain gas: 35 PSI; nebulizer gas: 55 PSI; auxiliary gas: 55 PSI; declustering potential: 80V (+) and 80V (−); collision energy: 10 eV (+) and −10 eV (−); interface heater temperature: 550°C (+) and 550°C (−). The LC-MS data were processed using Progenesis QI V2.3 (Nonlinear, Dynamics, Newcastle, UK) for baseline filtering, peak identification, integration, retention time correction, peak alignment, normalization, and analysis.

### Drug administration and experimental protocol

The mice were acclimatized for one week before the experiment. Subsequently, they were randomly assigned to the following eight groups: control group, sham group (sham operation), sham + empty bottle stimulation group, model group (combination of ovariectomy and empty bottle stimulation), positive control group (Progynova, 0.13 mg/kg, Bayer HealthCare), YHT low-dose group (YHT-L, 5.85 g/kg), YHT middle-dose group (YHT-M, 11.7 g/kg), and YHT high-dose group (YHT-H, 23.4 g/kg) (10). All drugs were administrated daily *via* the intragastric route. The mice in the control group did not undergo any intervention throughout the experiments.

After the habituation period, mice in the sham + empty bottle stimulation, model, Progynova, YHT-L, YHT-M, and YHT-H groups were trained to drink water at a specific time for 14 days. Furthermore, mice in these groups (except the sham + empty bottle stimulation group) underwent bilateral ovariectomy on the 11th day. From the 15^th^ to the 35^th^ day, the mice in these groups underwent empty bottle stimulation. On the 36^th^ day, all the animals were sacrificed for further analysis.

### Bilateral ovariectomy

This model is described in detail in our previously published study (11). Briefly, mice were anesthetized, and the operation area was shaved and disinfected using 75% ethanol. A small incision (1-1.2 cm) was then made along the ventral midline between the midpoint of the leading edge of the pubic bone and the navel. The uterus and the side of the uterine horn were found, and the ovary was identified along the uterine horn. Subsequently, the ovarian mesenteric blood vessels were ligated with silk threads, and the ovaries were removed with a pair of small forceps and scissors. Finally, the incision was closed with sutures.

### Empty bottle stimulation

Empty bottle stimulation was then used to create a model of chronic emotional stress (11). For two weeks, mice were trained to drink water from 08:00 h to 08:10 h and 20:00 h to 20:10 h by allowing them access to water bottles only during these time periods. To induce emotional stress, mice were given empty water bottles irregularly during one of the two watering periods for three weeks after training. Mice in the control and sham groups were provided water ad libitum throughout the experiment.

### Body weight and organ index evaluation

All mice were weighed once per week throughout the experiment. After three weeks of treatment, all mice were sacrificed, and the uteri, adrenal glands, spleens, thymi, and pituitary glands were carefully dissected, washed with cold sterile physiological saline, and weighed. Their weights relative to their final body weights were calculated as organ indices. After weighing, the uteri and vaginas were fixed with 10% neutral buffered formalin solution for subsequent histological assessment.

### Histopathological assessment

Hematoxylin and eosin (H&E) staining was performed for the histological assessment. The uteri and vaginas were fixed with 10% neutral buffered formalin solution, embedded in paraffin wax, and stained with H&E in 5-µm sections using a standard protocol. All images were captured using a microscope (ECLIPSE 50i, Nikon, Japan). The white balance was adjusted for all images.

### Pentobarbital-induced sleep test

The pentobarbital-induced sleep test was performed with a slight modification to a previous report (12). Briefly, mice were administered with pentobarbital sodium (50 mg/kg, i.p.) to induce sleep. The latency of the loss of the righting reflex after pentobarbital administration (“onset” time) and the total sleeping time (the time between the loss and the recovery of the righting reflex, “duration”) were determined for each mouse. The mouse was considered awake if it could right itself (return to the upright position). The time between the loss and recovery of the righting reflex was defined as sleep time. The entire process of the test was recorded using a digital video recorder for analysis.

### Behavioral analysis

Behavioral assessments were performed as described in our previous study (11). The mice were tested in a cage with only an empty bottle. The behavior of each mouse was recorded using a digital video recorder for 10 min. The observed behaviors were divided into three patterns: attacking behavior (biting or attacking the empty bottle and cage), exploring behavior (moving around or frequently visiting the place of the bottle), and grooming behavior (combing fur or touching the face by themselves). Each behavioral pattern was counted 10 times during the 10-minutes testing (once per minute). Once an animal exhibited a particular pattern of the three patterns, the score of that pattern was recorded as 1; otherwise, it was recorded as 0. The score for each behavioral pattern ranged from 0 to 10. The analysis was performed by an independent investigator who was blinded to group assignments.

### Enzyme linked immunosorbent assay

Serum estradiol levels were measured using a commercially available enzyme-linked immunosorbent assay (ELISA) kit. The ELISA kit for mouse estradiol was purchased from Nanjing Jiancheng Bioengineering Institute (catalog no. E-20380, Nanjing, China), and used following the manufacturer’s instructions.

### Network pharmacology analysis

The target proteins of each ingredient in YHT were obtained from the Traditional Chinese Medicine Systems Pharmacology Database and Analysis Platform (TCMSP) (13). Menopause-related targets were collected from the GeneCards database (14). The targets of YHT and menopause were introduced into Venny 2.1.0 to identify overlapping targets. Afterward, the ingredients-targets-disease network and protein-protein interaction (PPI) network were constructed using the Cytoscape V3.9.0 software. Gene Ontology analysis (including biological processes, cellular components, and molecular functions) was performed using the Database for Annotation, Visualization, and Integrated Discovery (DAVID) and Bioinformatics online tool (www.bioinformatics.com.cn). The functions of common genes were analyzed using the Metascape database (15).

### Molecular docking

We first obtained the three-dimensional structures of ingredients and target proteins from the TCMSP and Protein Data Bank (PDB) databases, respectively (16). Water molecules and ligands of the target proteins were removed using PyMOL software. Next, the target proteins were hydrogenated and converted to PDBQT format. AutoDock software was used to obtain molecular docking results. Finally, the PyMOL software was used to visualize the results.

### Statistical analysis

Data were presented as means ± standard errors of the mean. The mean number of data in individual groups was compared using the one-way/two-way analysis of variance (ANOVA) followed by Tukey’s test *post-hoc* analysis. Statistical work was performed using SPSS version 17.0 and a p-value < 0.05 was considered statistically significant.

## Results

### Compounds characterization of YHT

**Figure 1:**
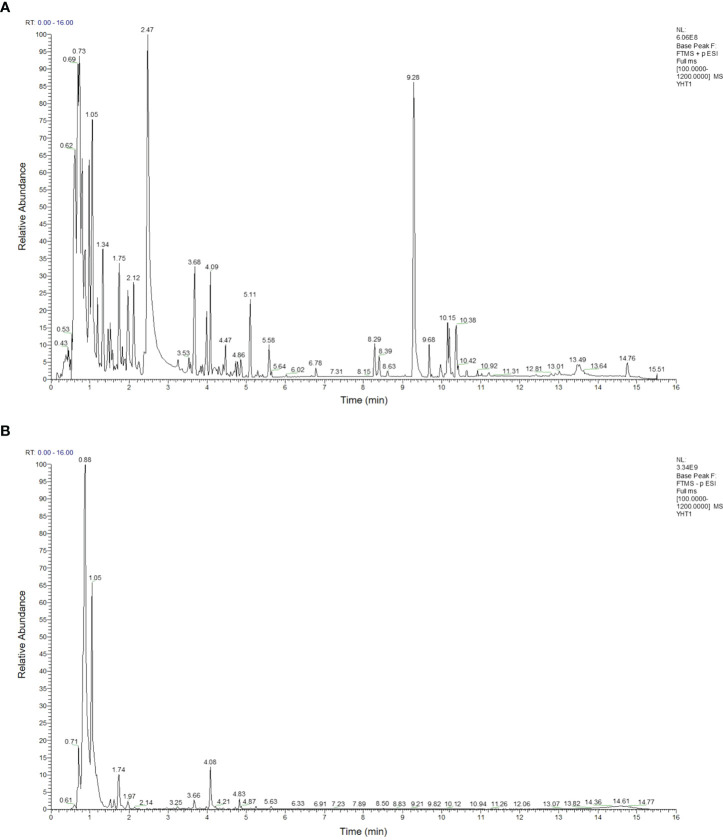
Base peak intensity chromatograms of YHT acquired by LC-MS. **(A)** Positive mode. **(B)** Negative mode.

As shown in **Table 2**, 93 compounds were characterized using LC-MS, including galactitol, betaine, glucosamine, and L-asparagine. The base peaks in the positive and negative ion modes from the YHT analysis are presented in **Figure 1**. Among the 93 compounds, 56 were detected in positive ion mode and 37 in negative ion mode. For further study, the TCMSP database was used to predict the corresponding potential targets of the 93 compounds.

**Table 2:** Identified chemical compounds within YHT by LC-MS.

No.	RT (min)	Ion mode	Identification	Score	Error (ppm)	KEGG ID	Formula
1	0.71	neg	Galactitol	47.3	-3.60	C01697	C6H14O6
2	0.71	pos	Betaine	40.3	-0.06	C00719	C5H11NO2
3	0.71	pos	Glucosamine	50.7	-0.55	C00329	C6H13NO5
4	0.71	pos	L-Asparagine	53.5	-0.11	C00152	C4H8N2O3
5	0.73	pos	Sucrose	53.1	-0.58	C00089	C12H22O11
6	0.75	pos	Creatine	38.8	0.31	C00300	C4H9N3O2
7	0.75	pos	L-Proline	57.4	1.61	C00148	C5H9NO2
8	0.78	neg	Maleic acid	37.4	-7.12	C01384	C4H4O4
9	0.84	pos	Adenine	46.3	-0.10	C00147	C5H5N5
10	0.87	pos	Citric acid	43.3	-0.38	C00158	C6H8O7
11	0.87	pos	Guanine	54.5	-0.23	C00242	C5H5N5O
12	1.05	neg	Uridine	54.2	0.14	C00299	C9H12N2O6
13	1.06	pos	L-Tyrosine	48.7	0.38	C00082	C9H11NO3
14	1.16	pos	L-Lysine	38.7	-0.21	C00047	C6H14N2O2
15	1.16	neg	Quinic acid	51.5	1.30	C00296	C7H12O6
16	1.24	neg	Guanosine	52.5	1.33	C00387	C10H13N5O5
17	1.27	neg	Aucubin	51.7	1.54	C09771	C15H22O9
18	1.33	pos	Adenosine	57.2	0.32	C00212	C10H13N5O4
19	1.35	neg	Ascorbic acid	47.6	-3.83	C01041	C6H8O6
20	1.40	pos	Phenylethylamine	37.5	3.37	C05332	C8H11N
21	1.50	neg	Fumaric acid	39.7	0.07	C00122	C4H4O4
22	1.55	pos	Hippuric acid	44.5	0.59	C01586	C9H9NO3
23	1.61	pos	Hordenine	44.5	0.29	C06199	C10H15NO
24	1.63	neg	Gallic acid	56.5	-3.81	C01424	C7H6O5
25	2.02	neg	Kojic acid	46.2	-2.71	C14516	C6H6O4
26	2.11	pos	Hydroquinone	44.8	2.84	C00530	C6H6O2
27	2.11	pos	L-Phenylalanine	54.5	0.33	C00079	C9H11NO2
28	2.22	neg	Shikimic acid	48.4	-4.29	C00493	C7H10O5
29	2.24	pos	Biotin	43.3	1.20	C00120	C10H16N2O3S
30	2.25	neg	Catalpol	52.1	2.18	C09773	C15H22O10
31	2.31	neg	Benzoic acid	37.9	-5.07	C00539	C7H6O2
32	2.68	pos	4-Methylcatechol	38.9	2.43	C06730	C7H8O2
33	2.93	neg	Chlorogenic acid	54	1.90	C00852	C16H18O9
34	2.95	neg	(-)-Epigallocatechin	51.6	1.63	C12136	C15H14O7
35	3.05	pos	Pimelic acid	39.1	0.02	C02656	C7H12O4
36	3.42	pos	Gentisic acid	39.2	0.48	C00628	C7H6O4
37	3.48	pos	Genistein	36.5	1.16	C06563	C15H10O5
38	3.58	neg	L-Tryptophan	53.8	-1.58	C00078	C11H12N2O2
39	3.62	pos	Ephedrine	38.9	0.72	C01575	C10H15NO
40	3.62	pos	Daidzin	37.6	0.14	C10216	C21H20O9
41	3.72	neg	Aconitic acid	46.2	-3.57	C00417	C6H6O6
42	3.76	pos	Epicatechin	46.3	-0.13	C09727	C15H14O6
43	3.83	pos	Piperine	36.7	1.33	C03882	C17H19NO3
44	3.87	neg	Protocatechuic acid	48.1	-5.19	C00230	C7H6O4
45	3.89	neg	trans-Cinnamic acid	38.7	-2.99	C10438	C9H8O2
46	3.89	pos	p-Anisic acid	45.6	0.40	C02519	C8H8O3
47	3.95	pos	Cinnamic acid	42.7	0.48	C10438	C9H8O2
48	3.99	pos	Citraconic acid	38.7	-0.18	C02226	C5H6O4
49	4.02	pos	Isoferulic acid	54.1	0.25	C10470	C10H10O4
50	4.03	neg	Trehalose	38.8	-0.13	C01083	C12H22O11
51	4.08	pos	Naringin	38.7	1.28	C09789	C27H32O14
52	4.11	neg	Geniposidic acid	55.4	1.34	C11673	C16H22O10
53	4.12	pos	4-Hydroxybenzoic acid	39.3	1.39	C00156	C7H6O3
54	4.15	neg	Isomangiferin	45.1	2.84	C16979	C19H18O11
55	4.18	neg	Scopoletin	50.5	-2.84	C01752	C10H8O4
56	4.22	pos	Apigenin	41.6	-0.43	C01477	C15H10O5
57	4.26	neg	Caffeic acid	56.6	-3.83	C01481	C9H8O4
58	4.33	pos	Phenylacetaldehyde	53.3	2.11	C00601	C8H8O
59	4.36	neg	Harpagoside	41.1	2.05	C09783	C24H30O11
60	4.37	pos	Coniferin	39.1	-1.04	C00761	C16H22O8
61	4.41	pos	Sebacic acid	39.2	-0.16	C08277	C10H18O4
62	4.48	neg	Syringic acid	56.2	-2.52	C10833	C9H10O5
63	4.49	pos	3-O-Feruloylquinic acid	50.4	-0.20	C02572	C17H20O9
64	4.74	neg	Suberic acid	53.3	-3.71	C08278	C8H14O4
65	4.82	neg	Methyl jasmonate	38.9	1.63	C11512	C13H20O3
66	4.82	pos	Vanillin	54.2	-0.44	C00755	C8H8O3
67	4.84	pos	Tyramine	39	0.73	C00483	C8H11NO
68	4.94	pos	Quercitrin	57	-0.31	C01750	C21H20O11
69	5.03	neg	Isorhamnetin	54	1.80	C10084	C16H12O7
70	5.04	pos	Nobiletin	38.7	-1.32	C10112	C21H22O8
71	5.06	pos	Papaverine	36.4	-0.62	C06533	C20H21NO4
72	5.12	neg	Biochanin A	39.1	1.63	C00814	C16H12O5
73	5.18	neg	Azelaic acid	55.4	-1.24	C08261	C9H16O4
74	5.40	neg	Cinnamaldehyde	38.7	-4.53	C00903	C9H8O
75	5.42	pos	Ononin	57.3	-1.21	C10509	C22H22O9
76	5.42	pos	Hesperetin	37.9	-0.68	C01709	C16H14O6
77	5.42	neg	Salicylic acid	55	-7.04	C00805	C7H6O3
78	5.82	neg	Formononetin	57.3	0.87	C00858	C16H12O4
79	5.88	pos	Kaempferol	38.7	-1.27	C05903	C15H10O6
80	5.91	neg	Quercetin	51.5	1.01	C00389	C15H10O7
81	5.94	neg	Vanillic acid	52.1	-4.42	C06672	C8H8O4
82	6.10	pos	Catechol	38.8	1.82	C15571	C6H6O2
83	6.14	pos	Protopine	38.8	-1.20	C05189	C20H19NO5
84	6.41	pos	Glutaric acid	38.8	0.04	C00489	C5H8O4
85	6.54	pos	Sclareol	37	-0.78	C09183	C20H36O2
86	7.01	pos	Sinapic acid	39.6	-0.16	C00482	C11H12O5
87	7.56	neg	Scoparone	46.5	1.18	C09311	C11H10O4
88	8.63	pos	Curdione	42.7	-0.85	C17493	C15H24O2
89	9.77	pos	Gamma-Linolenic acid	39	0.16	C06426	C18H30O2
90	9.85	pos	Alpha-Linolenic acid	39.3	-0.24	C06427	C18H30O2
91	10.17	pos	Estradiol	50.2	-0.13	C00951	C18H24O2
92	11.20	pos	Dibutyl phthalate	44.3	-0.17	C14214	C16H22O4
93	11.93	pos	Oleic acid	39.5	-0.07	C00712	C18H34O2

RT, retention time; pos, positive; neg, negative.

### Effects of YHT on body weight and organ index in menopausal mice

**Figure 2:**
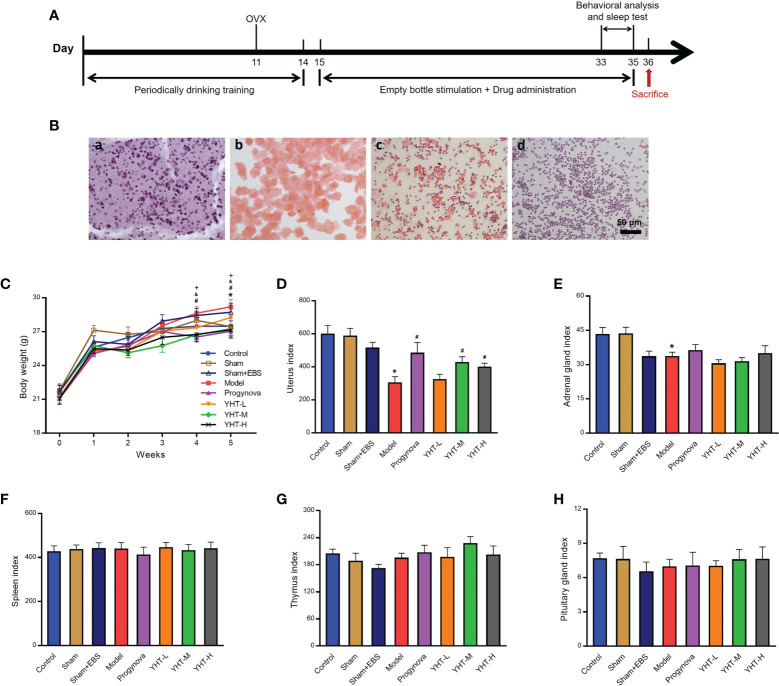
Effects of YHT on the body weight and organ index in menopausal mice. **(A)** Experimental manipulation of mice during the study. **(B)** H&E staining of vaginal smears from mice in the four different phases of the estrous cycle. a) Proestrus. b) Estrus. c) Metestrus. d) Diestrus. **(C)** Body weight of mice in 5 weeks. *p < 0.05, control group vs model group; #p < 0.05 progynova group vs model group; &p < 0.05 YHT-M group vs model group; **^+^** p < 0.05 YHT-H group vs model group. **(D–H)** Organ index of the uterus, adrenal gland, spleen, thymus, and pituitary. *p < 0.05 vs control group; #p < 0.05 vs model group. EBS, empty bottle stimulation.

The experimental workflow used in this study is shown in **Figure 2A**. The estrous cycle stages were identified based on the cell types present, as shown in **Figure 2B**. Mice in the control group presented different stages of the estrous cycle. Mice in the ovariectomy groups only presented the diestrus stage, which is characterized by a large number of leukocytes (shown in **Figures 2B–D**). Body weight and organ indices were measured to identify the effects of YHT on the mice. The results showed that the body weight at week five was significantly higher in the model group than in the control group (**Figure 2C**). Compared with the model group, the body weights at week four and week five were significantly lower in the progynova, YHT-M, and YHT-H groups (**Figure 2C**). No significant difference in body weight was observed between the model and YHT-L groups throughout the experiment. Additionally, the organ index of the uterus was lower in the model group than that in the control group (**Figure 2D**). The uterine index in the progynova, YHT-M, and YHT-H groups were higher than that in the model group (**Figure 2D**). The adrenal gland index was significantly lower in the model group than that in the control group (**Figure 2E**). Neither progynova nor YHT reversed the decrease in adrenal gland index in menopausal mice. Our results also showed that the organ indices of the spleen, thymus, and pituitary gland did not show any significant difference in any of the groups (**Figures 2F–H**).

### Histological effects of YHT on uterus and vagina in menopausal mice

**Figure 3:**
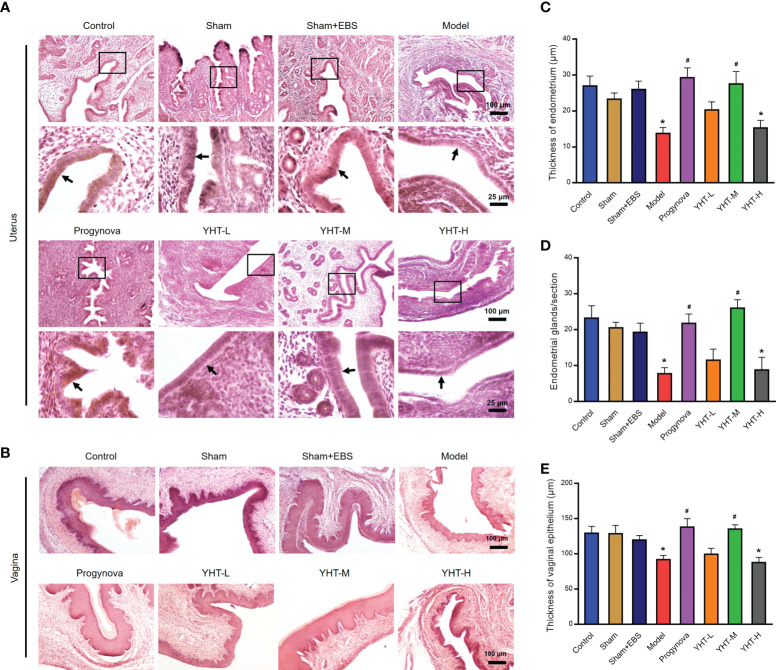
Histological effects of YHT on the uterus and vagina in menopausal mice. **(A)** Uterus. **(B)** Vagina. **(C)** Thickness of endometrium. **(D)** Number of endometrial glands. **(E)** Thickness of vaginal epithelium. *p < 0.05 vs control group; #p < 0.05 vs model group. EBS, empty bottle stimulation.

To identify the effects of YHT on organ structure, the histomorphology of the uterus and vagina was assessed using H&E staining (**Figures 3A, B**). In menopausal mice, the lining of the uterus (endometrium) became thinner and smoother, and the endometrial glands decreased significantly compared to the control mice (**Figures 3C, D**). The histomorphology of the uterus in the Progynova, YHT-M, and YHT-H groups was similar to that of the control group. However, in the YHT-L group, the histomorphology of the uterus did not change much compared to the model group (**Figures 3C, D**). Compared to the control group, the vaginal epithelium was atrophied in the model group (**Figure 3E**). In the Progynova and YHT-M groups, vaginal epithelium atrophy was reversed. However, no significant difference in the histomorphology of the vagina was observed between the YHT-L and YHT-H groups and the model group (**Figure 3E**).

### Effects of YHT on anxiety-like behavior and sleeping behavior in menopausal mice

**Figure 4:**
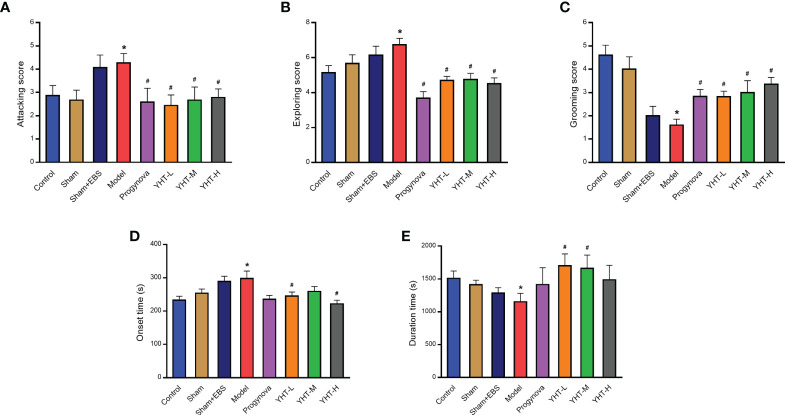
Effects of YHT on sleeping behavior and anxiety-like behavior in menopausal mice. **(A)** Attacking behavior. **(B)** Exploring behavior. **(C)** Grooming behavior. **(D)** Onset time of sleep. **(E)** Duration time of sleep. *p < 0.05 vs control group; #p < 0.05 vs model group. EBS, empty bottle stimulation.

Mice in the model group exhibited significant attacking behaviors compared with mice in the control group (**Figure 4A**). However, compared with the model group, attacking behaviors in all other groups were significantly decreased. In the model group, the mice demonstrated more exploratory behaviors than that in the control group (**Figure 4B**). However, the other groups showed less exploratory behaviors compared to the model group. In addition, grooming behaviors in the model group were reduced compared with those in the control group (**Figure 4C**). However, grooming behaviors in the other groups were greater than those in the model group.

To examine the effects of YHT on sleep behavior, all the mice were injected with pentobarbital (50 mg/kg). The results demonstrated that the onset time in the model group was longer than that in the control group (**Figure 4D**). In the YHT-L and YHT-H groups, the onset time decreased significantly compared to that in the model group. However, no significant difference was found in the onset time between the Progynova and YHT-M groups and the model group (**Figure 4D**). Moreover, compared to the control group, the model group exhibited a significant decrease in duration (**Figure 4E**). The duration increased greatly in the YHT-L and YHT-M groups compared with that in the model group. However, the duration in the Progynova and YHT-H groups was not significantly different from that in the model group (**Figure 4E**).

### Ingredients-targets-disease network construction and PPI analysis

**Figure 5:**
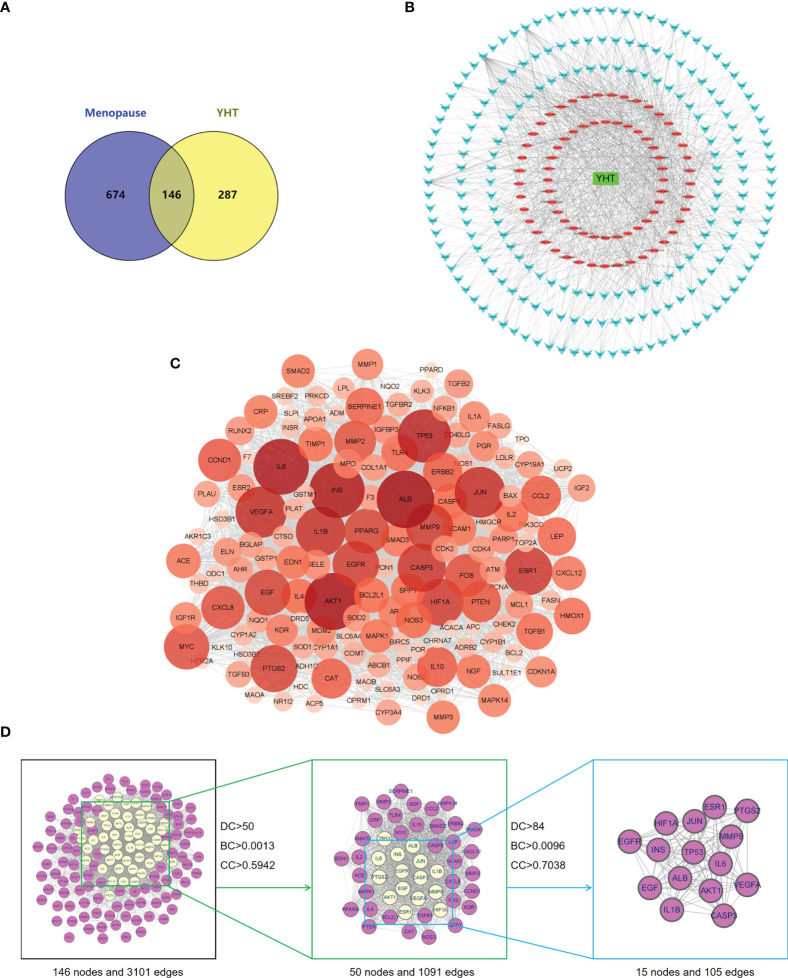
The ingredients-targets-disease network and PPI network analyses of YHT. **(A)** Venn diagram of YHT targets and menopause-related targets. **(B)** The ingredients-targets-disease network of YHT. **(C)** The PPI network diagram of targets. **(D)** The network topology analysis of the PPI network.

Using the TCMSP database, we identified 433 potential targets of YHT. Next, we predicted a total of 820 menopause-related targets from the GeneCards database (Relevance score > 1.00). After overlap, 146 intersectional genes were identified as potential key targets of YHT in the treatment of menopause (**Figure 5A**). The ingredients-targets-disease network was then constructed by using Cytoscape V3.9.0 software (**Figure 5B**). This network includes 274 nodes and 1129 edges. YHT active ingredients are represented by orange nodes, menopause targets by blue nodes, and YHT itself by green nodes. The number of links connected to other nodes indicates the importance of a node in the network (17). Topological analysis of degree revealed that the top 10 active ingredients were: quercetin, genistein, kaempferol, apigenin, estradiol, isorhamnetin, formononetin, nobiletin, epicatechin, and maleic acid.

The PPI network included 146 nodes and 3101 edges, and the average number of neighbors was 42.5 (**Figure 5C**). Topological analysis revealed that the top 10 core targets in the network were: ALB, AKT1, IL6, INS, TP53, VEGFA, JUN, IL1B, ESR1, and CASP3. Network topology analysis of the PPI network is shown in **Figure 5D**. Core targets were obtained by filtering twice with scores for degree centrality (DC), betweenness centrality (BC), and closeness centrality (CC).

### GO and pathways enrichment analysis

**Figure 6:**
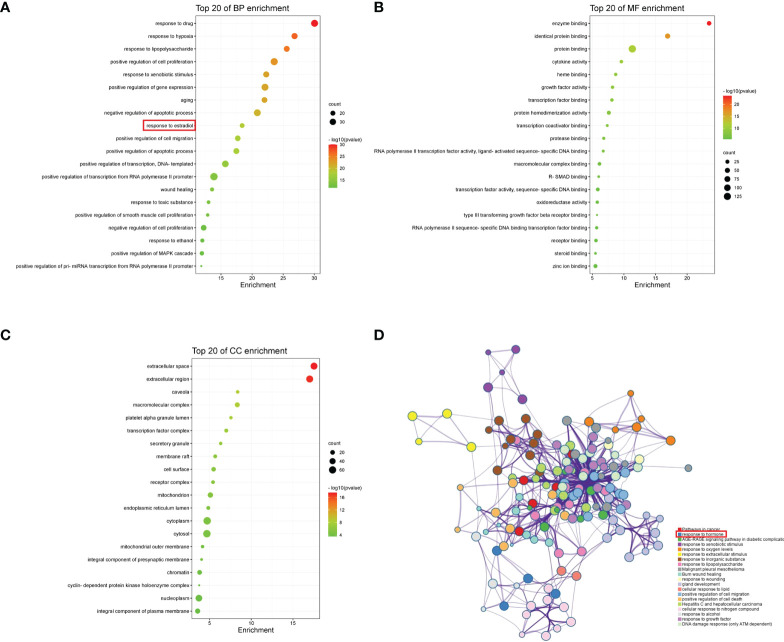
GO and pathway enrichment analyses of YHT. **(A)** Biological process. **(B)** Molecular function. **(C)** Cellular component. **(D)** Network of enriched pathways.

We obtained 1006 GO items from the DAVID, including 782 biological process (BP), 79 cellular component (CC), and 145 molecular function (MF) terms. The top 20 most enriched items are shown in a bar chart (**Figures 6A–C**). The BPs were mainly related to the response to hypoxia, positive regulation of cell proliferation, aging, negative regulation of the apoptotic process, and response to estradiol. CCs were mainly related to the extracellular space, extracellular region, caveola, macromolecular complex, and platelet alpha granule lumen. The MFs were mainly related to enzyme binding, protein binding, cytokine activity, and heme binding. Pathway enrichment analysis revealed that the most significantly enriched pathways were cancer, response to hormones, and AGE-RAGE signaling pathways (**Figure 6D**).

### Molecular docking analysis

**Figure 7:**
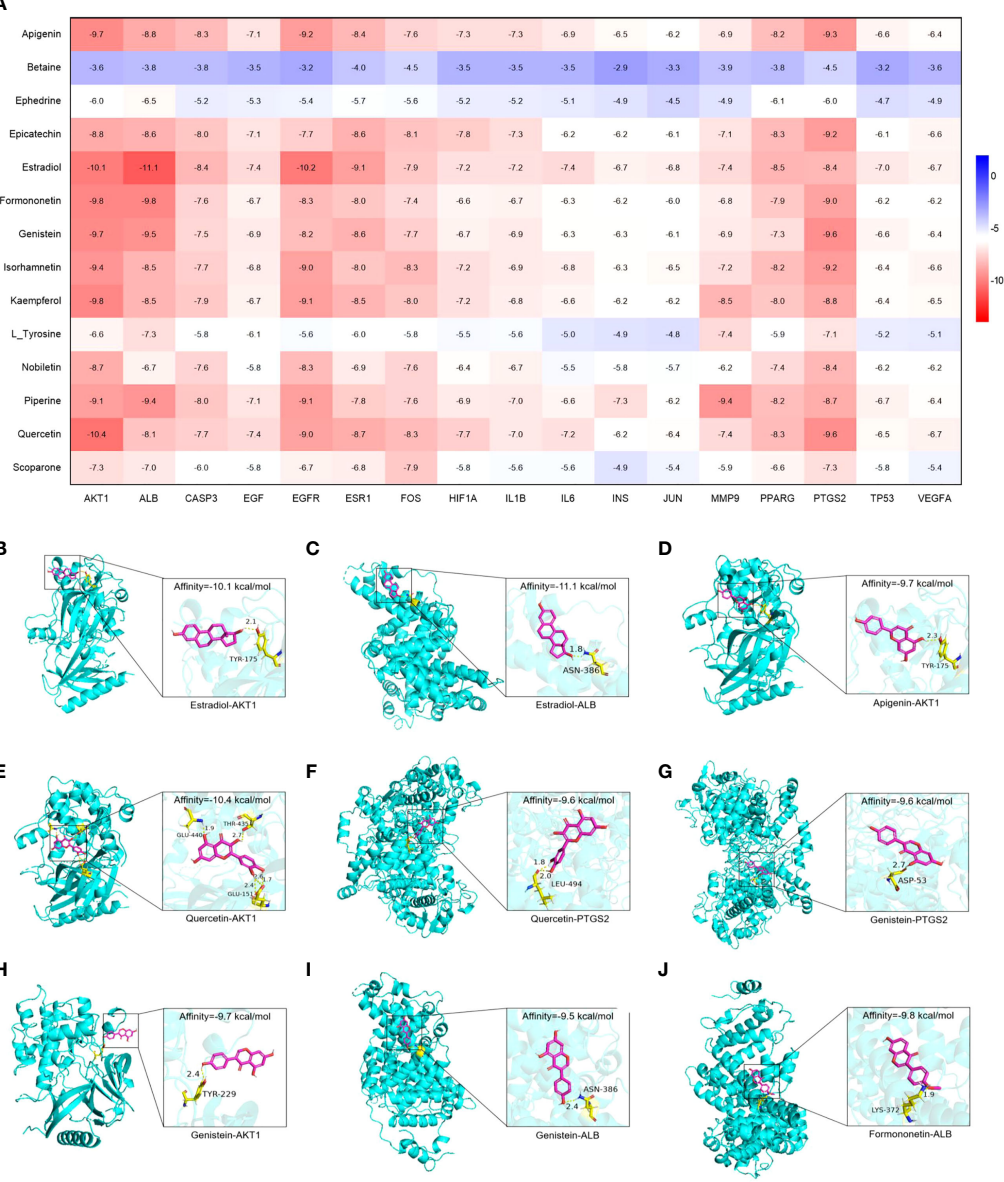
Molecular docking analysis of the main compounds and key targets. **(A)** Molecular docking heatmap. **(B)** Estradiol-AKT1. **(C)** Estradiol-ALB. **(D)** Apigenin-AKT1. **(E)** Quercetin-AKT1. **(F)** Quercetin-PTGS2. **(G)** Genistein-PTGS2. **(H)** Genistein-AKT1. **(I)** Genistein-ALB. **(J)** Formononetin-ALB.

Molecular docking was used to evaluate the binding abilities of the core compounds and protein targets of YHT. The lower the binding affinity, the better the binding activity between the compound and protein (18). As shown in **Figure 7**, AKT1, ALB, EGFR, ESR1, and PTGS2 generally exhibited good docking performance with the core active compounds of YHT (apigenin, estradiol, formononetin, genistein, and quercetin). In particular, Estradiol showed a high binding affinity of −11.1 kcal/mol and −10.2 kcal/mol in ALB and EGFR, respectively. Quercetin showed a high binding affinity of −10.4 kcal/mol in AKT1. Interestingly, estradiol showed a high binding affinity of −9.1 kcal/mol in ESR1, which further confirmed the potential key role of estradiol-related pathways.

### Experimental validation and potential pathways prediction

**Figure 8:**
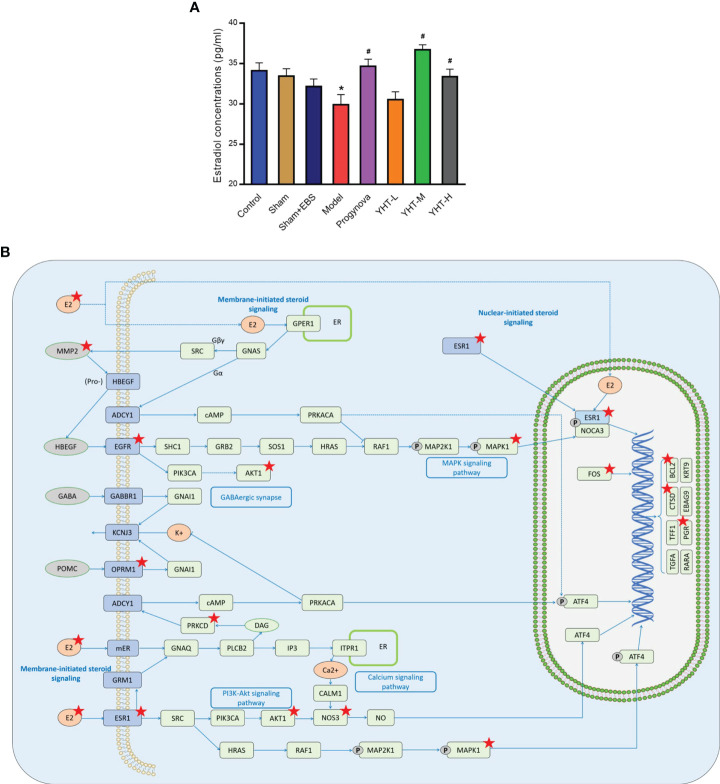
Effects of YHT on the serum estradiol concentrations in menopausal mice. **(A)** Serum estradiol concentrations. *p < 0.05 vs control group; #p < 0.05 vs model group. **(B)** The estradiol signaling pathway. EBS, empty bottle stimulation.

To validate the results of network pharmacology and molecular docking, we measured serum levels of estradiol. The estradiol concentration in the model group was lower than that in the control group (**Figure 8A**). However, estradiol concentrations in the Progynova, YHT-M, and YHT-H groups were significantly higher than those in the model group. No significant differences were observed between the YHT-L and model groups (**Figure 8A**). Finally, the KEGG PATHWAY database was used to predict the potential pathways involved in the effects of YHT. According to the above analysis, the estradiol signaling pathway is considered to be a critical pathway underlying the protective effects of YHT (**Figure 8B**). Multiple molecules, such as ESR1, MAPK1, and AKT1, may participate in the therapeutic mechanism of YHT.

## Discussion

Several women experience a range of menopause-like symptoms related to changes in sex hormone levels during menopause (19). The most common symptoms include depressive disorders, sleep disturbances, sexual dysfunction, muscle pain, osteoporosis, and characteristic hot flashes (20). HRT is believed to be the most effective intervention for menopausal symptoms (21). However, recent evidence has shown that long-term HRT may have negative consequences on women’s health (22, 23). In this study, bilateral ovariectomy and empty bottle stimulation were combined to induce menopause-like symptoms in the mice. Our study provides evidence that YHT administration in animals partly relieved menopause-like symptoms and provides a rationale for using the YHT as a potential therapeutic strategy to ameliorate menopausal syndrome.

YHT has already been used clinically to treat menopausal symptoms by many TCM doctors, with good effects (6, 7). This ancient herbal formula was created by Chen Shiduo, an outstanding physician during the Qing dynasty in China. According to TCM theory, YHT was applied to treat the syndrome of hyperactivity due to Yin deficiency, and symptoms of this syndrome were similar to female menopausal symptoms. Therefore, it is theoretically feasible to treat the menopausal symptoms of YHT. In addition, progynova was selected as the positive control agent because it showed a potent effect in many menopausal animal studies (24, 25).

The ovariectomy-induced menopausal model has been widely used in many studies. Empty bottle stimulation was initially used to induce an emotional stress model (26). Animals that underwent empty bottle stimulation showed a state of irritability, which was similar to the clinical situation of patients with an anxiety disorder (26, 27). In our previous study, we combined ovariectomy and empty bottle stimulation to induce menopause-like symptoms in mice (28). This model expressed typical menopause-like symptoms such as reduced estrogen level and sleep quality, increased body weight, and aggressive behaviors.

Being overweight and obese causes not only the physical inconvenience but also secondary diseases such as diabetes, coronary heart disease, hypertension, and stroke (29). The risk of overweight or obesity induced by estrogen deficiency increases significantly in post-menopausal females (30). In our study, the body weight of menopausal mice increased significantly compared to that of control mice. However, YHT showed an obvious improvement in weight gain. Given that estrogen plays an important role in body weight regulation, this mechanism may be related to an increase in estrogen levels (31). Menopause results in loss of ovarian function and an atrophic state of genital organs, such as the uterus and vagina (32). The reduction in the uterine index due to ovariectomy was reversed by YHT. In addition, the adrenal gland, spleen, thymus, and pituitary gland indices did not change in this study. The morphological results were consistent with those of the uterine index. YHT exhibited good effects in inhibiting endometrial atrophy and vaginal epithelial atrophy induced by ovariectomy and empty bottle stimulation.

Sleep disturbances are common during menopause (33). Menopausal women frequently complain of having difficulty in sleep initiation or sleep maintenance (34). However, the mechanisms underlying sleep disturbances that arise during menopause remain unclear. In this study, YHT significantly shortened the process of falling asleep and increased the duration of continuous sleep. However, based on our results, progynova did not improve sleep disturbance induced by ovariectomy and empty bottle stimulation. This suggests that purely supplemented estradiol did not present sufficient efficacy to relieve sleep disturbances during menopause.

With the decline in ovarian steroid levels, the peri- and postmenopausal periods represent a window of vulnerability for the emergence of anxiety symptoms and disorders (35). Many women experience profound anxiety attacks that start occurring while experiencing menopausal symptoms. The connection between anxiety attacks and menopause remains clear. Mice showed significant anxiety-like behaviors after ovariectomy and empty-bottle stimulation, and anxiety was significantly attenuated by YHT. Current data indicate that YHT may be as effective as estradiol in relieving anxiety during menopause.

Estrogens are female sex hormones involved in many physiological processes, especially reproductive development and function. Estradiol, the main form of circulating estrogen, is secreted by ovaries (36). The decline of estradiol at menopause often leads to functional disorders that affect the quality of life (37). In our study, YHT reversed the decline in estradiol induced by menopausal syndrome. This may partly explain why YHT relieved the atrophy of sexual organs and anxiety.

We obtained 93 active ingredients of YHT using LC-MS analysis. Among them, quercetin, genistein, kaempferol, apigenin, and estradiol were considered the core ingredients. Quercetin has been reported to increase the antioxidant capacity of ovaries in menopausal rats and *in vitro* (38). Genistein exhibits estrogenic activity and improves uterine morphology in mice (39). Another study demonstrated that kaempferol might exert a protective effect against post-menopausal bone loss (40). Recently, a study revealed that apigenin alleviates xerostomia *via* estrogen receptor α-mediated upregulation of aquaporin 5 activation (41). PPI analysis showed that ALB, AKT1, IL6, and INS were the key targets of YHT in treating menopause-like symptoms in mice. ALB, AKT1, IL6, and INS indicate albumin, RAC-alpha serine/threonine-protein kinase, interleukin-6, and insulin, respectively. Early menopause is associated with higher risk of urinary albumin-creatinine ratio elevation in postmenopausal women (42). The expression of Akt1 dysregulated in menopausal rats (43). In addition, the increased deposition of visceral fat promotes the release of interleukin-6 in the postmenopause period (44). Increased inflammation of beta cells impairs insulin secretion in a post-menopausal diabetic rat model (45).

BP enrichment and pathway enrichment analysis showed that the estradiol signaling pathway might play a key role in the protective effects of YHT. The docking results also showed that estradiol and ESR1 had a good docking performance. The predicted results were verified by ELISA of the serum estradiol levels. We found that YHT reversed the decrease in serum estradiol levels in menopausal mice. Our results are similar to those of a previous study (46). In that study, YHT promoted the release of endogenous estrogen in the brain and improved depression-like behavior induced by chronic unpredictable mild stress in ovariectomized mice.

In this study, we explored the optimal dosage of YHT for attenuation of menopause-like symptoms in mice. Interestingly, we did not observe a distinct dose-dependent effect of YHT on physiological and psychological symptoms in mice with menopausal syndrome among the three experimental dosages used. However, we found that mice in the YHT middle-dose group showed more improvement than that in the low-dose group in some indicators (such as body weight, uterus index, endometrial thickness, gland number, the thickness of vaginal epithelium, and estradiol concentration). The effect of the middle dosage of YHT was found to be the best among the three doses tested, which was probably due to the toxicity of YHT at high dosages and suggests the importance of exploring the optimal dosage in the clinic setting (47).

Our study has some limitations that need to be discussed. First, this study investigated only the pharmacological properties of YHT in menopausal animals. Further research needed to investigate how YHT can improve menopausal syndrome. Second, network pharmacology technology only provides clues based on existing research results, which indicates that the innovativeness of this study is relatively limited. Omics technologies such as transcriptomics and proteomics are needed to explore the targets of YHT for treating menopause syndrome.

## Conclusion

In conclusion, YHT improved the bilateral ovariectomy and empty bottle stimulation-induced menopause-like symptoms in mice. Our findings suggest that YHT significantly ameliorates both physiological and psychological symptoms in a novel mouse model through a multitarget and multi-pathway approach. Animal experiments have validated that the estradiol-mediated pathway may play a key role in this process. Considering its significant therapeutic potency, we concluded that YHT has the potential to improve menopause-like symptoms in middle-aged and older women. Further studies on the potential mechanisms and compositional analysis of YHT are needed in the future.

## Data availability statement

The original contributions presented in the study are included in the article/**Supplementary Material**. Further inquiries can be directed to the corresponding authors.

## Ethics statement

The animal study was reviewed and approved by Committee on the Ethics of Heilongjiang University of Chinese Medicine.

## Author contributions

YY: data collection, data analysis, manuscript writing. BZ: data collection, data analysis. YL: data collection. H-DX: writing - review and editing. X-ML: writing - review and editing. S-MH: supervision. RW: conceptualization, supervision. DL: project administration. All authors contributed to the article and approved the submitted version.

## Funding

This work was supported by National Natural Science Foundation of China (No. 81873108, 82003975), Natural Science Foundation of Heilongjiang Province (No. H2015-068), Project of Qiqihar Academy of Medical Sciences (No. QMSI2020L-05), and Excellent Creative Talents Support Program of Heilongjiang University of Chinese Medicine (No. 2018RCQ08).

## Conflict of interest

The authors declare that the research was conducted in the absence of any commercial or financial relationships that could be construed as a potential conflict of interest.

## Publisher’s note

All claims expressed in this article are solely those of the authors and do not necessarily represent those of their affiliated organizations, or those of the publisher, the editors and the reviewers. Any product that may be evaluated in this article, or claim that may be made by its manufacturer, is not guaranteed or endorsed by the publisher.
