# Supplementary material for: Yin Huo Tang, a traditional Chinese herbal formula, relives ovariectomy and empty bottle stimulation-induced menopause-like symptoms in mice
Source: Front Endocrinol (Lausanne). 2022 Oct 19;13:994642. doi: 10.3389/fendo.2022.994642 (PMC9627159; doi:10.3389/fendo.2022.994642)
Supplement: Supplementary file 1 [file DataSheet_1.pdf]

Supplementary Materials

Yin Huo Tang, A Traditional Chinese Herbal Formula, Relives Ovariectomy and Empty Bottle Stimulation-induced Menopause-like Symptoms in Mice

Yang Ye[1†], Bo Zhang[2†], Yan Li[3], Hong-Dan Xu[4], Xiu-Min Liu[5], Shu-Ming Huang[2], Rui Wang[6*] and Dong Li[1*]

[1]Department of Traditional Chinese Medicine, Peking University Third Hospital, Beijing, China

[2]Department of Neuroscience, Institute for Chinese Medicine, Heilongjiang University of Chinese Medicine, Harbin, China

[3]Department of Integrated Traditional Chinese and Western Medicine, College of Medicine, Yangzhou University, Yangzhou, China

[4]Department of Pharmacy, Wuxi Higher Health Vocational Technology School, Wuxi, China

[5]National Institute on Drug Dependence and Beijing Key Laboratory of Drug Dependence, Peking University, Beijing, China

[6]Research Institute of Medicine and Pharmacy, Qiqihar Medical University, Qiqihar, China

***Correspondence:**

Rui Wang, wrdoctor1975@163.com

Dong Li, lidong6512@sina.com

[†]These authors have contributed equally to this work and share first authorship.

Graphical Abstract

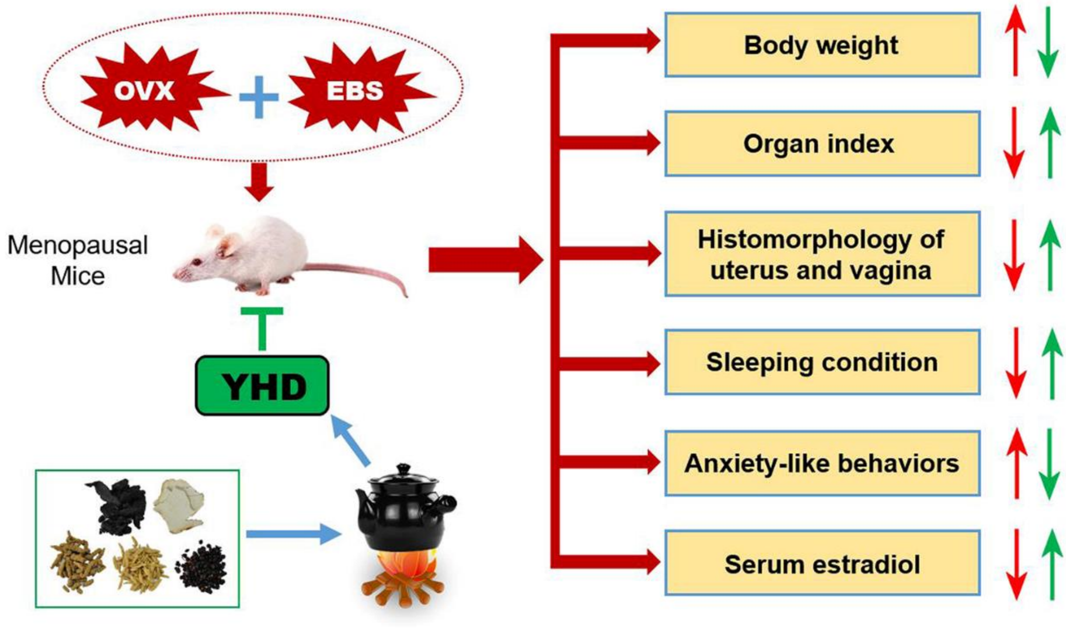

Supplementary Methods

Determination of Estrous Stage

The length of the estrous cycle in mice is about 5-7 days. Microscopic evaluation of the types of cells present in vaginal smears has long been applied to determine the stages of the estrous cycle in mice. Vaginal secretions were collected with a pipette filled with 15 μL of normal saline solution by inserting the tip into the mouse vagina. Then the vaginal wash was aspirated, and placed on a clean glass slide. Samples were subsequently stained with hematoxylin and eosin and examined under a microscope.
